# Excision of Extensive Orbitozygomaticomaxillary Complex Tumors Combining an Intra- and Extraoral Approach With Transpalpebral Orbital Exenteration

**DOI:** 10.3389/fvets.2020.569747

**Published:** 2020-12-11

**Authors:** Amy E. Thomson, Brittney E. Rigby, Alexander T. Geddes, Jason W. Soukup

**Affiliations:** Dentistry and Oromaxillofacial Surgery, Department of Surgical Sciences, School of Veterinary Medicine, University of Wisconsin-Madison, Madison, WI, United States

**Keywords:** maxillectomy, zygomectomy, zygomatic tumors, zygomaticomaxillary tumors, orbitozygomaticomaxillary complex tumors, oral tumors, maxillary tumors, orbitectomy

## Abstract

The junction of the bones of the orbit, caudal maxilla and zygoma intersect to form an anatomically intricate region known as the orbitozygomaticomaxillary complex (OZMC). Given the critical role of the OZMC in the structure, function and esthetics of the skull and midface, tumors in this region present unique challenges to the oromaxillofacial surgeon. Attempts to achieve histologically clean tumor margins in a cosmetically pleasing manner requires excellent intra-operative visualization. Additionally, minimized intra-operative and post-opertive complications is of paramount importance. In this manuscript we describe a combined intra- and extraoral approach to extensive tumors of the OZMC that incorporates orbital exenteration as a technique, which allows for excellent intra-operative visualization and mitigate intra- and post-operative complications. In addition, we describe our experience utilizing the technique in five clinical cases.

## Introduction

It is generally accepted that the primary goal of any tumor excision is to obtain tumor-free margins. Maintaining proper function, managing local pain, and achieving good to excellent cosmesis are additional goals. The degree to which one may achieve these goals is dependent on location and biological behavior of the tumor as well as the complexity of the resultant reconstruction procedure.

**Figure 1:**
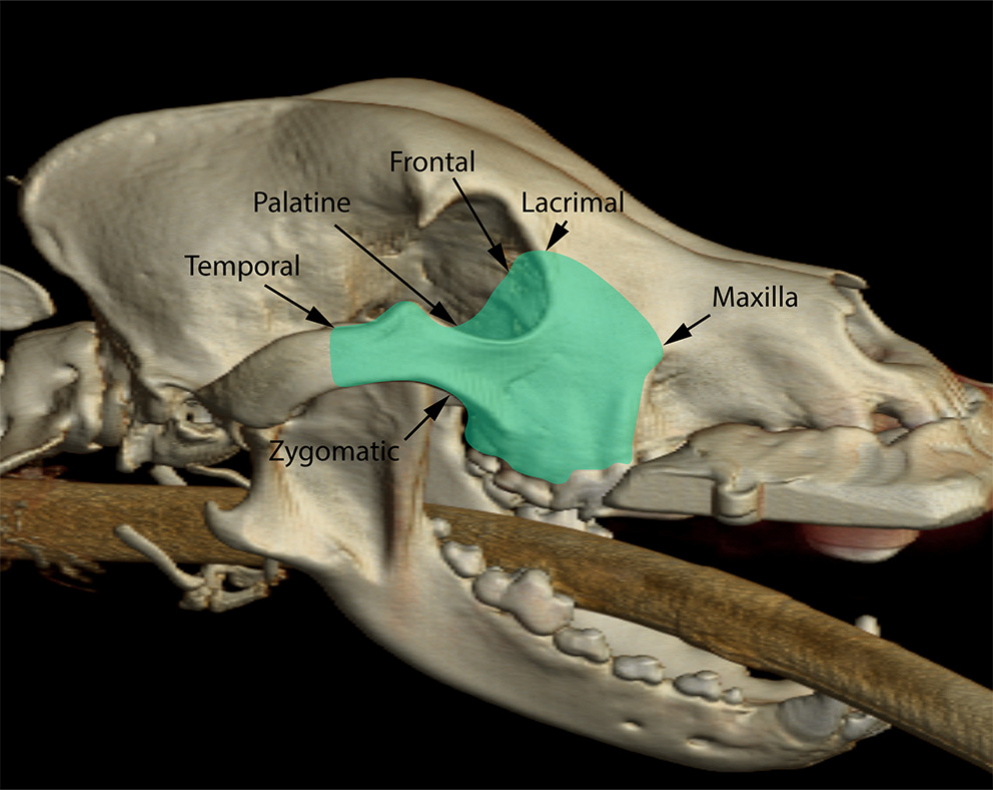
Three-dimensional rendering of a dog skull highlighting (green overlay) the various osseous components of the orbitozygomaticomaxillary complex.

The orbitozygomaticomaxillary complex (OZMC) is the region of the skull where the zygomatic, and maxilla bones as well as bones that contribute to the medial orbital wall (lacrimal, frontal, and palatine bones) unite (Figure 1). The OZMC plays a critical role in the structure, function and esthetics of the skull and orbit contributing to the strength, stability and normal anatomical contours of the facial skeleton. Given the unique intersection of structurally and cosmetically important structures; the proximity of the orbit to the calvarium; and the challenges of access, the surgical excision of OZMC tumors may present unique challenges that make primary surgical goals difficult to achieve.

Excision of OZMC tumors often involves the medial orbital wall, which requires excellent visualization to prevent inadvertent penetration of the rostral cranial fossa. Additionally, significant ophthalmic side-effects, such as enophthalmos, diplopia and entropion, are expected if orbital reconstruction is not pursued (1–5). The degree to which a tumor of the OZMC has invaded orbital and periorbital structures may be uncertain. In such situations, a surgical approach to OZMC tumors that combines an intra- and extraoral approach with transpalpebral exenteration may be utilized. Transpalpebral exenteration is a modification to a previously reported technique (6, 7) that facilitates surgical excision of tumors significantly involving the medial orbit. Here we describe this approach to OZMC tumors and report our clinical experience in five dogs. To the authors' knowledge, such an approach has not previously been described in the peer-reviewed English language literature.

## Surgical Technique

Prior to surgical planning all patients received a head computed tomography (CT) study with and without contrast (GE Lighspeed Ultra, GE Healthcare, Milwaukee, WI), mandibular lymph node aspiration as well as aspiration of any parotid or medial retropharyngeal nodes that showed deviation from normal size or echogenicity. The airway was secured by standard orotracheal intubation immediately following induction of general anesthesia. Due to the nature of an academic anesthesia and pain management service, anesthetic protocols were not standardized and were determined by the attending anesthesiologist. Where indicated, extraction of occluding mandibular teeth was preformed. In order to minimize oral bacterial load and contamination, an oral antiseptic (0.12% chlorhexidine) rinse was applied and the dentition in the region of the surgery were scaled and polished. Intra-operative regional anesthesia using 0.5% bupivicaine was provided with an ultrasound-guided trigeminal nerve block as described by Viscasillas et al. (8) Dogs were then positioned in a modified dorsal recumbency position with the head positioned slightly lateral. The entire ipsilateral maxilla and skull was clipped and prepared for aseptic surgery from the nasal planum caudally to the level of the vertical ear canal and from ventral midline of the lower jaw to at least 2 cm beyond the dorsal midline of the head and muzzle. A sterile surgical pen was used to outline the extraoral approach and the intraoral surgical margins of 1–2 cm based on the combination of tumor type, size and location; biological behavior of the tumor (as determined from history and pre-operative computed tomographic studies); client goals; and expected post-operative patient function and cosmesis. A temporary tarsorrhaphy was performed prior to beginning the surgical approach.

**Figure 2:**
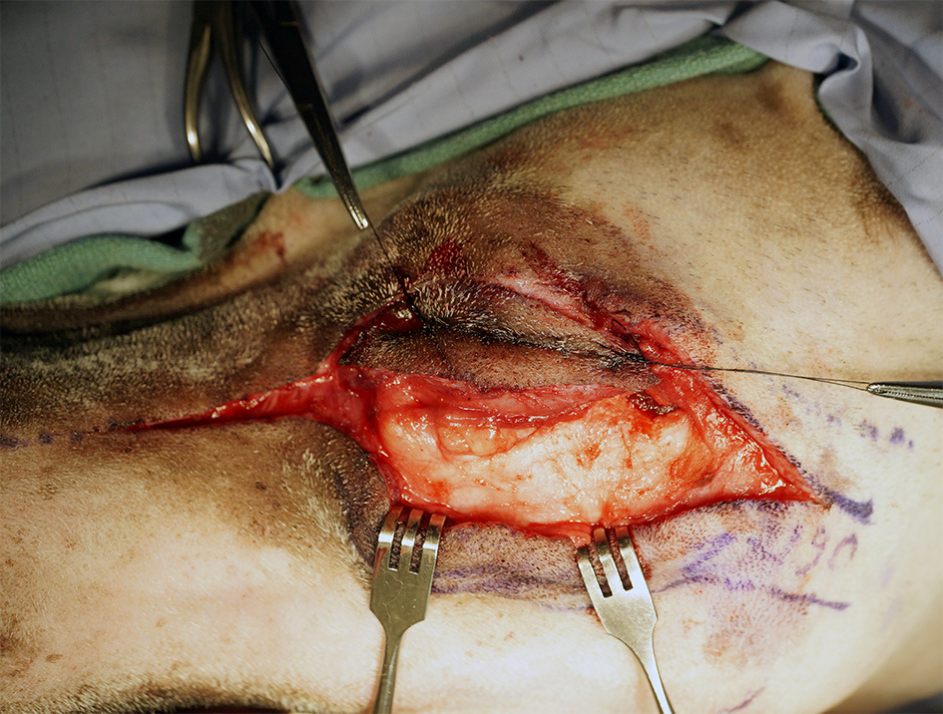
Intraoperative photograph depicting the extraoral approach combining a horizontal incision from the dorsolateral maxilla into the zygoma with a transpalpebral approach to orbital exenteration.

**Figure 3:**
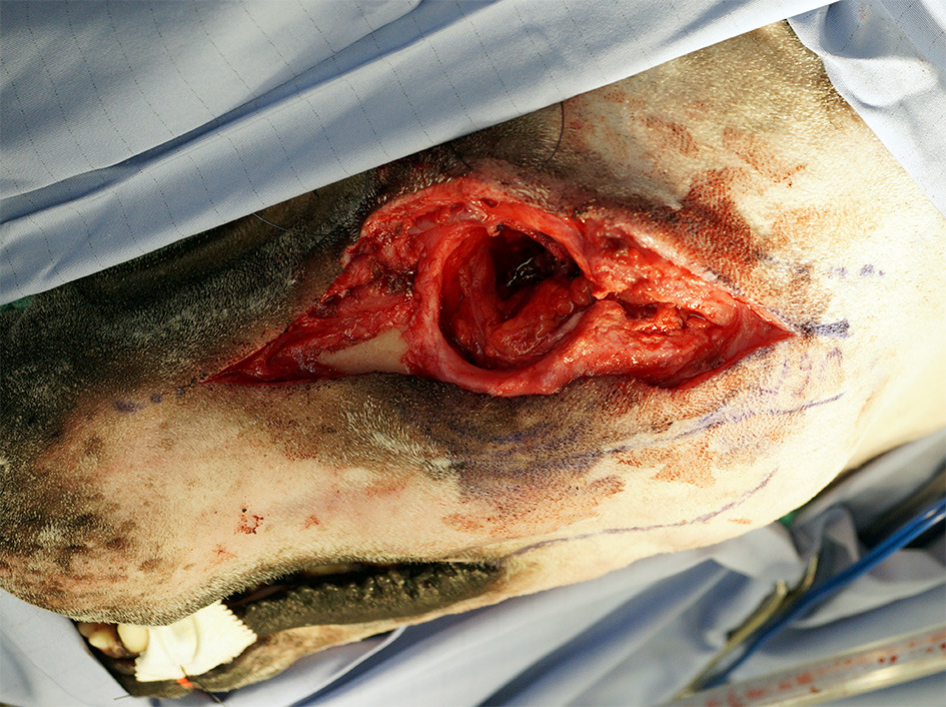
Intraoperative photograph depicting completion of the extraoral approach with orbital exenteration.

An extraoral incision was begun ~3 cm rostral to the medial canthus of the eye along the dorsolateral muzzle and extending caudally toward the medial canthus of the eye. The incision was continued caudally, incorporating a transpalpebral incision, and extending along the dorsal aspect of the zygoma (Figure 2). Exenteration of the globe was performed as described elsewhere (9) (Figure 3). Following exenteration, a commissurotomy was preformed to allow for improved visualization and access to the caudal oral cavity (10).

**Figure 4:**
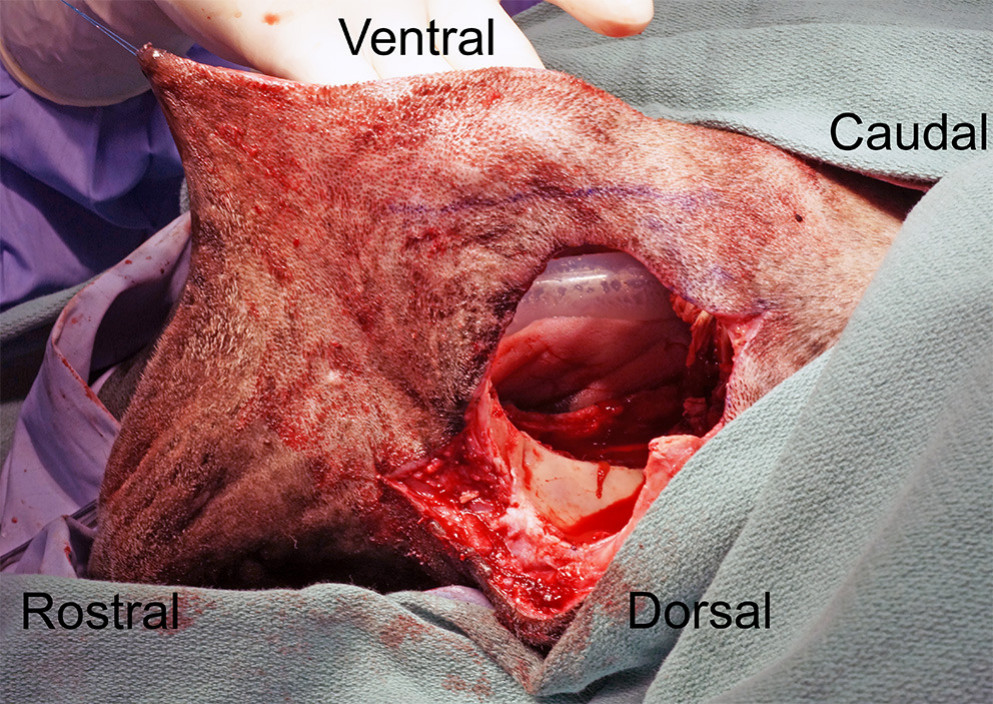
Intraoperative photograph depicting the communication of the intra- and extraoral approaches; in effect, creating a bipedicle flap of dermis and oral mucosa.

The intraoral approach was commenced by incising the gingiva, vestibular and hard palatal mucosa along the intraoral surgical markings. A combination of sharp and blunt dissection was used to incise the submucosal tissue and muscles along the maxilla and zygoma to approach the underlying OZMC. The levator nasolabialis, orbicularis oris, rector anguli oculi, zygomaticus, sphincter coli pars intermedia and frontalis muscles were incised followed by the levator labii maxillaris and caninus, masseter and temporalis muscles. Vessels crossing the surgical margins were double ligated with a 4-0 synthetic monofilament absorbable suture (Monocryl, Ethicon) and transected. A periosteal elevator was used to reflect the periosteum and associated soft tissues off the underlying bones. By joining the intraoral approach with the extraoral approach, this step, in effect, created a composite bipedicle flap (Figure 4).

**Figure 5:**
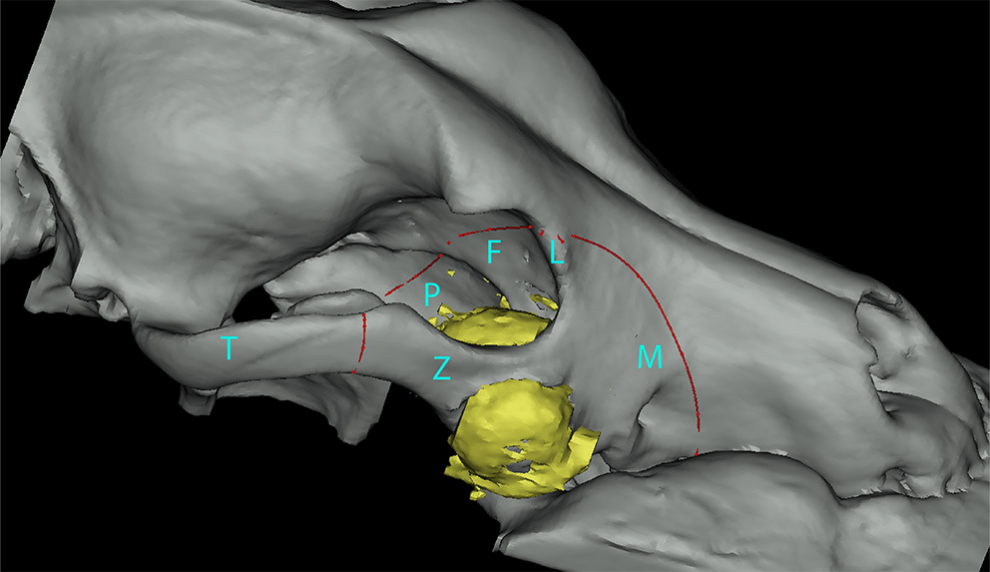
Segmented three-dimensional rendering of a dog maxilla representing an OZMC tumor (yellow) and the area of excision (red line). Bones of the OZMC are labeled (M, maxilla; L, lacrimal; F, frontal; P, palatine; Z, zygomatic; T, temporal).

**Figure 6:**
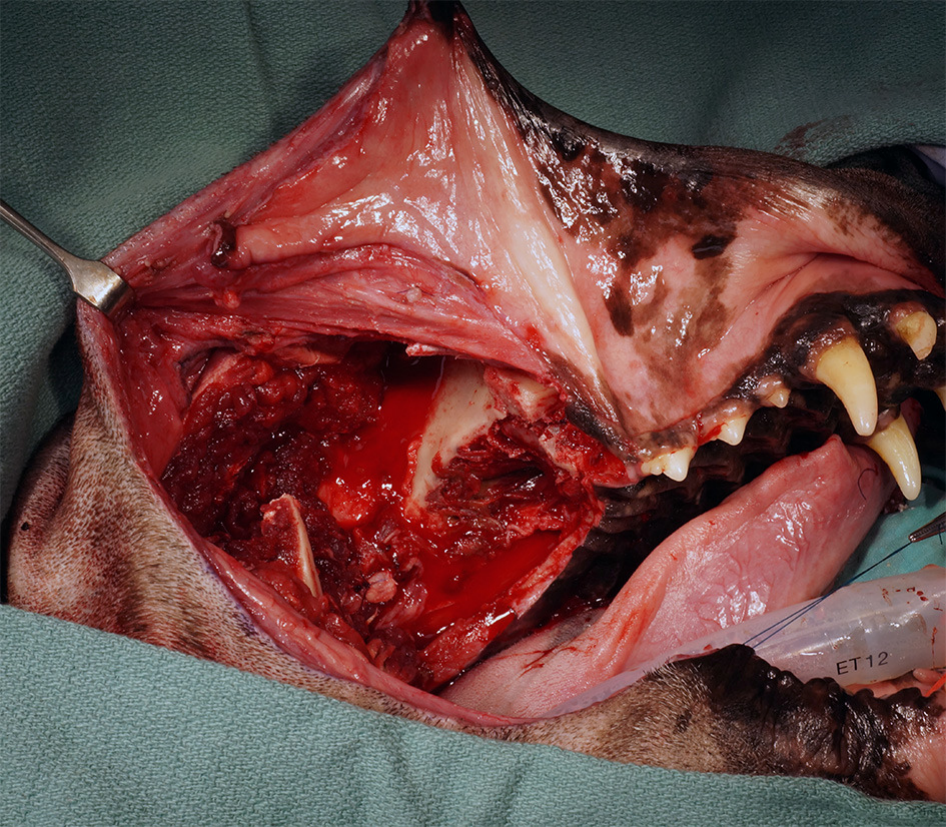
Intraoperative photograph depicting the intraoral view of the caudal maxilla after completion of the OZMC excision. Note a commissurotomy has been performed in this case to improve exposure.

**Figure 7:**
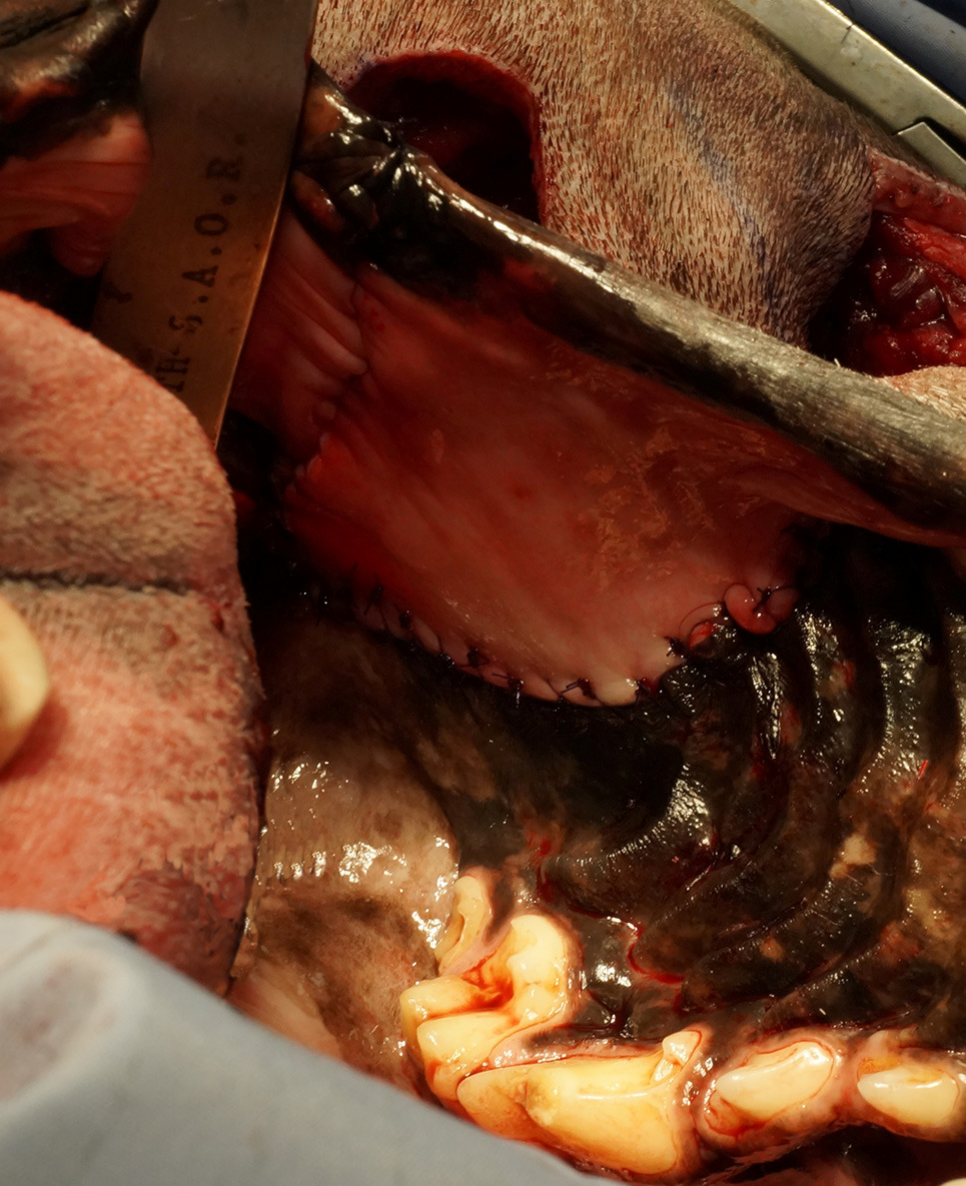
Intraoperative photograph depicting an intraoral view of the mucosal advancement flap for the vestibular reconstruction.

**Figure 8:**
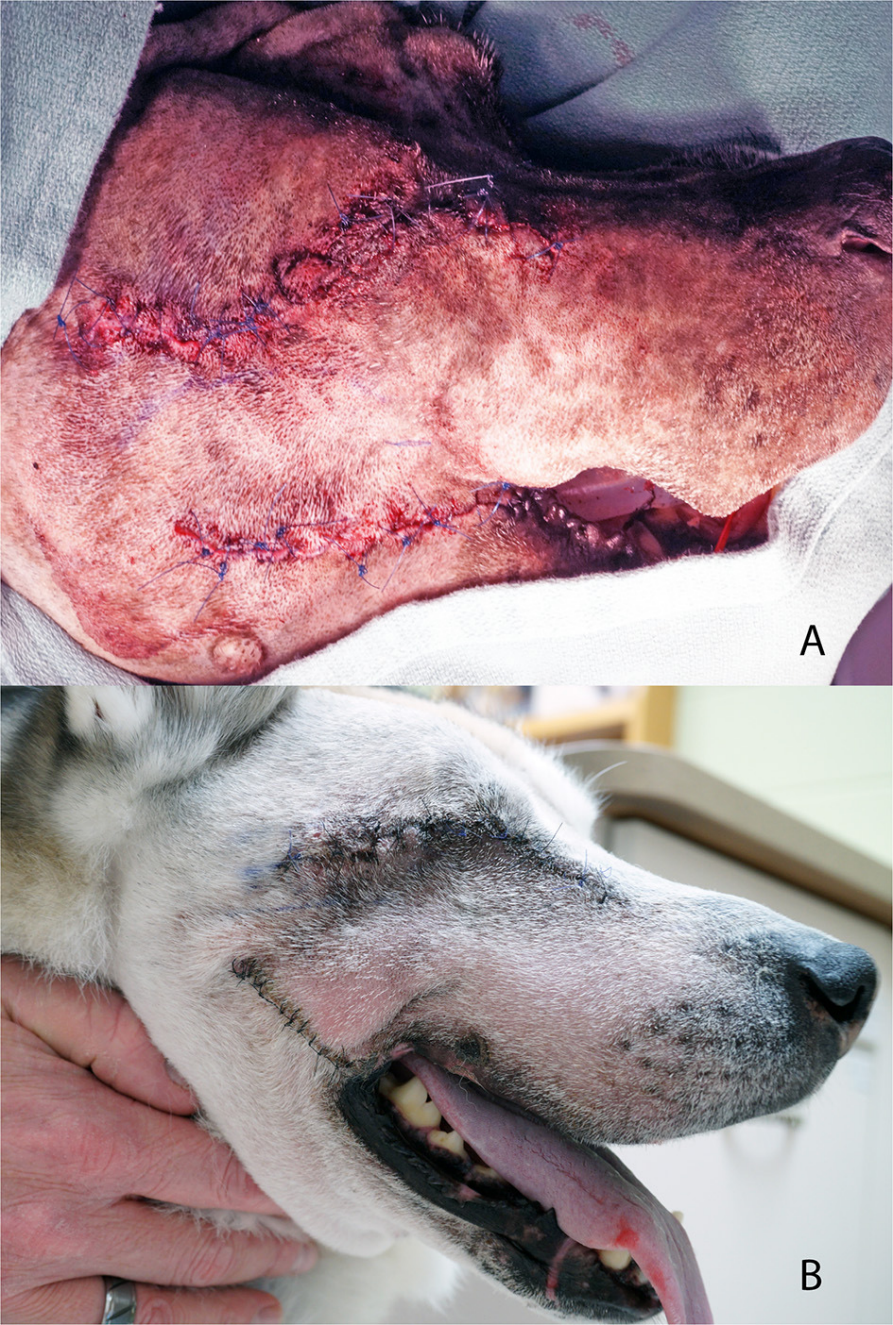
Photograph depicting an extraoral view of the dermal advancement flap and closure of a commissurotomy **(A)** and photograph of case #3 at the 14-day post-operative recheck examination **(B)**.

Utilizing a piezoelectric surgical unit, excision of the OZMC was performed by making osteotomies through the maxilla, palatine, lacrimal and zygomatic bones and the orbital part of the frontal bone and the zygomatic process of the temporal bones as well as the ethmoidal crest and sphenoethmodal plate, as dictated by the intended surgical margins (Figure 5). A winged dental elevator was then used to elevate the resected segment. The maxillary artery was then isolated and ligated with 4-0 synthetic monofilament absorbable suture (Monocryl, Ethicon). Nasal mucosa, ectoturbinates and endoturbinates were then incised with Metzenbaum scissors to complete the *en bloc* excision, which included the suborbital tissues and the zygomatic salivary gland when indicated (Figure 6). Prior to closure, a single injection of *Nocita*, a bupivacaine liposome injectable suspension, (0.4 ml/kg or 5.3 mg/kg) was infused into the muscular and subcutaneous tissues surrounding the excision to provide post-operative analgesia. The remaining bipedicle flap of the cheek/superior labia was then bluntly dissected to separate the buccal/labial mucosa from the dermis and muscles. An advancement flap was utilized to suture the buccal/labial mucosa to the palatal mucoperiosteum with 4-0 synthetic monofilament absorbable suture (Biosyn, Covidien) in a simple interrupted pattern (Figure 7). Extraorally, the muscles were apposed starting rostrally with the levator nasolabialis. Continuing with musculature closure caudally the orbicularis oris was apposed to the sphincter coli pars intermedia, followed by the rector anguli oculi and frontalis muscles apposed to the zygomaticus with 4-0 synthetic monofilament absorbable suture (Biosyn, Covidien) in a simple interrupted pattern. The dermis of the bipedicle flap was advanced dorsally and secured with 4-0 synthetic monofilament non-absorbable suture (Dermilon, Covidien) in a simple interrupted pattern (Figure 8). All patients remained in the critical care unit (CCU) following anesthesia to ensure adequate post-operative analgesia with hourly pain scoring. Instructions to feed only canned or softened kibble and to avoid toys or mouth play were given to the owners.

## Case Reports

### Case 1

A 10.5-year-old neutered male vizsla was referred to the Medical Oncology service for evaluation of a mass located on the palatal aspect of the right maxillary fourth premolar tooth. On presentation, the visible mass measured 1 cm in diameter, was erythematous with a smooth contour. Mandibular & medial retropharyngeal lymph node aspiration and thoracic radiography revealed no indication of metastasis. Computed tomography (CT) of the head revealed a lobulated, soft tissue attenuating, non-uniformly enhancing 1.6 cm diameter mass with numerous areas of patchy, coalescing mineralization along the palatal aspect of maxillary fourth premolar and molars. The tumor primarily involved the caudal maxilla and palatine bones; advancing dorsally into the pterygopalatine fossa. Previously perform histopathology revealed a diagnosis of osteosarcoma.

The case was referred to the Dentistry and Oromaxillofacial Surgery service for surgical excision. The tumor was excised as an en bloc excision of the OZMC and associated musculature, oral mucosa, gingiva and dentition utilizing a combined intra- and extraoral approach with a transpalpebral exenteration. The osseous excision extended from the mesial aspect of the right maxillary third premolar tooth to the mid-zygoma. The medial extent of the excision was the median palatine raphe and the inferior aspect of the medial orbit. Following excision, the wound was closed as described above.

Histological evaluation of the resected tissue confirmed the diagnosis of osteosarcoma and surgical margins were free of tumor with a narrow margin (16 mm) at the distal aspect of the excision. The dog recovered uneventfully from general anesthesia despite hypotension that responded to a transfusion of packed red blood cells. The patient remained on a continuous rate infusion (CRI) of fentanyl (3 μg/kg/hr), and ketamine (3 μg/min/hr) overnight. Transdermal fentanyl patches (100 mcg/hr + 25 mcg/hr) and ampicillin/sulbactam (20 mg/kg IV q 8 h) was also administered. The dog was discharged the following day with oral carprofen (2 mg/kg q 12 h), tramadol (5 mg/kg q 8–12 h) and amoxicillin/clavulanic acid (13.75 mg/kg q 12 h). Instructions to feed only canned or softened kibble and to avoid toys or mouth play were given to the owners.

At the time of recheck and suture removal 14 days later the owners reported the dog was eating and drinking well. The skin and oral incisions had healed well and no discharge, redness, swelling or dehiscence was noted. The owners elected to pursue definitive radiation therapy over 20 fractions of 2.7Gy to a total of 54Gy. This intensity-modulated radiation therapy was delivered using 6 MV helical tomotherapy (TomoTherapy HiArt Treatment System®, Accuray Inc., Sunnyvale, CA, USA) and planned with inverse planning software (TomoTherapy HiArt® v3-5)]. Following radiation therapy, vinorelbine chemotherapy was initiated. Pulmonary metastasis was identified 21 months after the surgical excision, radiation therapy and chemotherapy was initiated. Two weeks later, the patient was euthanized due to poor quality of life secondary to pulmonary disease.

### Case 2

**Figure 9:**
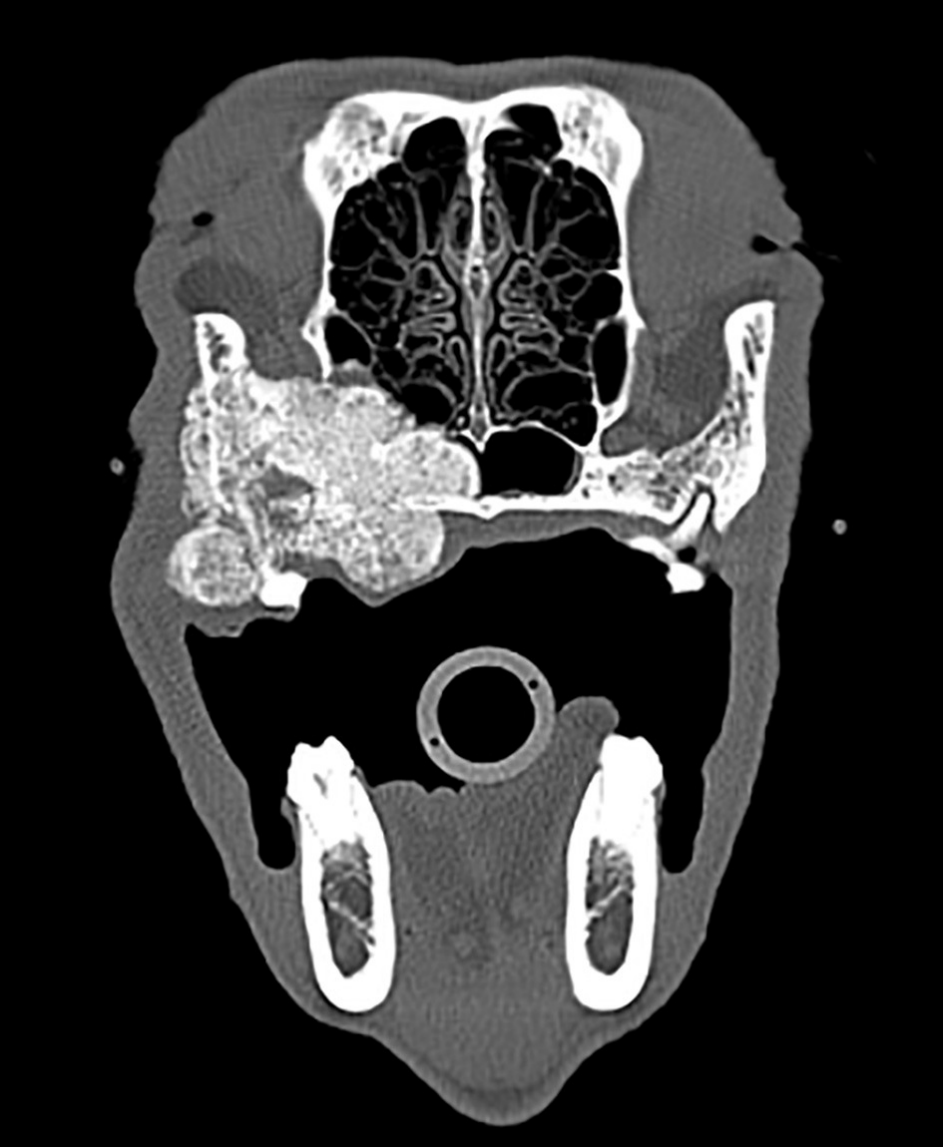
Axial CT image of a multilobular tumor of bone seen in case #2, which revealed a large lobulated, heterogenous, mineral attenuating 5.1 × 3.8 × 2.9 cm mass centered at the right maxillary fourth premolar tooth and first molar tooth.

A 7.5-year-old neutered male Standard Poodle was referred to the Dentistry and Oromaxillofacial Surgery service for evaluation and biopsy of an oral mass affecting the right caudal maxilla. An approximately 3.5 cm diameter, firm mass was present on the palatal aspect of the right maxillary fourth premolar tooth and first molar tooth. Thoracic CT, mandibular and medial retropharyngeal lymph node aspirations revealed no evidence of metastasis. Head CT revealed a large lobulated, heterogenous, mineral attenuating 5.1 × 3.8 × 2.9 cm mass centered at the right maxillary fourth premolar tooth and first molar tooth. The mass was invading and destroying the palatine, zygomatic and frontal bone as well as the rostroventral ethmoid turbinates, maxillary recess, infraorbital canal and nasopharyngeal meatus (Figure 9). Histopathology of the biopsied tissue revealed a diagnosis of multilobular tumor of bone.

The tumor was excised as an en bloc excision of the OZMC and associated musculature, oral mucosa, gingiva and dentition utilizing a combined intra- and extraoral approach with a transpalpebral exenteration. The osseous excision extended from the mesial aspect of the right maxillary third premolar tooth to the mid-zygoma. The medial extent was 7 mm toward the contralateral side from the median palatine raphe and included portions of the maxilla, lacrimal, palatine, pterygoid and frontal bones. Following excision, the wound was closed as described above.

Histological evaluation of the resected tissue confirmed the diagnosis of multilobular tumor of bone and tumor-free margins. All margins were reported as clean with at least 1 cm tumor-free tissue, except for the palatal margin, which was reported as narrow (3 mm). The dog recovered uneventfully from general anesthesia with minimal blood loss and was managed overnight on a CRI of sufentanil (0.3 μg/kg/hr), lidocaine (10 μg/min/hr) and ketamine (3 μg/min/hr). Additionally, a subcutaneous dose of carprofen (4.4 mg/kg) was administered. The dog was discharged the following day with a 150 mcg transdermal fentanyl patch, oral tramadol (4 mg/kg q 8-12 h) and carprofen (2.2 mg/kg q 12 h). The owners were instructed to feed only canned or softened kibble and to avoid toys or mouth play.

At seven days post-operatively there was significant halitosis noted by owners and evidence of a 3 cm x 12 cm dehiscence at the caudal aspect of the intraoral closure site. The remaining closure sites were healing well and a good cosmetic appearance was apparent. An island axial pattern flap based on the contralateral major palatine artery was used to close the caudal dehiscence and healing was uncomplicated. At 4 months post-operatively recurrence of the neoplasia was noted on CT and owners elected against further surgical intervention. The patient was palliatively managed with the referring veterinarian and euthanized 11 months after the original surgery date.

### Case 3

An 8.5-year-old neutered male Siberian husky was referred to the Dentistry and Oromaxillofacial Surgery service for treatment of a left maxillary papillary squamous cell carcinoma. On presentation, a 0.5 cm diameter, round and firm mass was visible in the buccal gingiva of the left maxillary first molar tooth. The patient was placed under general anesthesia for thoracic CT and regional lymph node aspiration, which revealed reactive lymphoid tissue and no evidence of pulmonary metastasis. A head CT was performed and revealed a 1.9 × 3.0 × 2.6 cm soft tissue attenuating, inhomogeneous contrast enhancing nodular mass centered at the left maxillary first molar tooth.

The tumor was resected as an en bloc excision of the OZMC and associated musculature, oral mucosa, gingiva and dentition utilizing a combined intra- and extraoral approach with a transpalpebral exenteration. The excision extended from the mesial aspect of the left maxillary fourth premolar tooth distally to the mid-zygoma. The palatal extent of the excision was the median palatine raphe. The excision extended from the inferior orbital crest of the lacrimal bone and maxilla at its dorsomedial extent to the dentition at its ventrolateral extent including the maxilla and dorsal zygoma. Following excision, the wound was closed as described above.

Histological evaluation of the resected tissue confirmed the diagnosis of papillary squamous cell carcinoma and surgical margins were free of tumor with a narrow margin (4 mm) at the distal aspect of the excision. While this patient received a whole blood transfusion due to hypotension in the face of only moderate blood loss, the dog recovered uneventfully from general anesthesia. The patient was managed overnight on a CRI of fentanyl (3 μg/kg/hr) and ketamine (3 μg/min/hr). Carprofen (2.2 mg/kg SQ) and cefazolin (25 mg/kg IV q 8 h) was also administered. The dog was discharged the following day with a 75 mcg transdermal fentanyl patch and oral carprofen (2.2 mg/kg q 12 h), tramadol (5 mg/kg q 8–12 h) and amoxicillin/clavulanic acid (13.75 mg/kg q 12 h). Instructions to feed only canned or softened kibble and to avoid toys or mouth play were given to the owners.

At a 14-day post-operative recheck examination the patient was doing well with occasional sneezing. The extraoral closure had healed well. The intraoral incision was mostly healed with only a pin-point (~0.5 mm) dehiscence seen at the rostral extent. At the next recheck under general anesthesia another 14 days later, persistence of the pin-point oronasal fistula previously noted was identified. Surgical correction was declined by the owners, as the patient was asymptomatic. Two weeks later, the patient presented for left nasal discharge with suspected rhinitis suspected to be due to the persistent pin-point oronasal fistula. Medical management was initiated with azithromycin (10 mg/kg q 24 PO x 2 weeks, then 10 mg/kg q48 PO × 2 weeks). At the last contact with owners, five months post-operative, the patient was doing well with no sneezing or nasal discharge and no gross evidence of recurrence.

### Case 4

**Figure 10:**
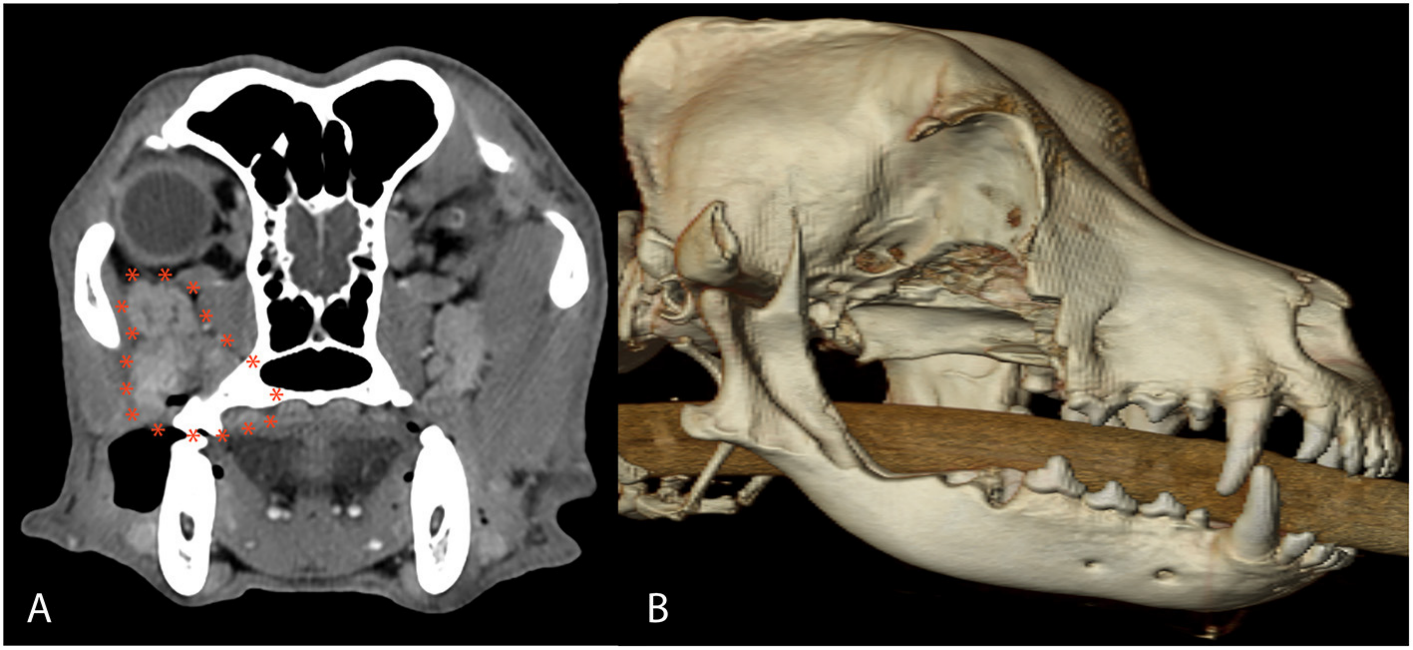
Axial CT image **(A)** and postoperative 3D CT image **(B)** in case #4. The axial CT reveals a 3 cm × 2 cm soft tissue attenuating mass along the right caudal maxilla extending from the distal aspect of the right maxillary second molar tooth and into the suborbital adipose tissues with enlarged and attenuating zygomatic salivary gland diagnosed as a spindle cell sarcoma. The 3D CT image depicts the extent of the OZMC excision, which included portions of the caudal maxilla, lacrimal, frontal, palatine, zygomatic, temporal, pterygoid, and mandible bones.

A 13-year-old neutered male Labrador retriever was referred to the Radiation Oncology service for evaluation and treatment of a suborbital spindle cell sarcoma. On presentation, a 1.5 cm diameter, multilobulated mass could be appreciated intraorally at the palatal aspect of the right maxillary second molar tooth. Thoracic radiography and regional mandibular lymph node aspiration was performed prior to referral, which revealed reactive lymphoid tissue and no evidence of pulmonary metastasis or intrathoracic lymphadenopathy. Head CT revealed a 3 cm x 2 cm soft tissue attenuating mass along the right caudal maxilla extending from the distal aspect of the right maxillary second molar tooth and into the suborbital adipose tissues with enlarged and attenuating zygomatic salivary gland (Figure 10).

The patient was referred to the Dentistry and Oromaxillofacial Surgery service for excision of the OZMC and associated musculature, oral mucosa, gingiva and dentition utilizing a combined intra- and extraoral approach with a transpalpebral exenteration. The excision extended from the furcation of the right maxillary fourth premolar tooth and extended caudally to the mid-zygoma. From dorsal to ventral, the excision extended from the inferior orbital crest of the lacrimal bone and extended into the mandibular ramus and body just ventral to the mandibular second and third molar teeth. The excision including portions of the caudal maxilla, lacrimal, frontal, palatine, zygomatic, temporal, pterygoid and mandible bones as well as the zygomatic salivary gland. The palatal extent of the excision was the median palatine raphe. Following excision, the mesial and palatal roots of the maxillary fourth premolar tooth were extracted and the wound was closed as described above.

Histological evaluation of the resected tissue confirmed the diagnosis of spindle cell sarcoma, but could not differentiate the tumor between fibrosarcoma and nerve sheath sarcoma. Greater than 1 cm tumor free histologic margins were reported. The dog recovered uneventfully from general anesthesia without any need for blood products and was managed overnight in the CCU on hydromorphone (0.5 mg/kg q 6 h IV). Meloxicam (0.2 mg/kg SQ) and ampicillin (22 mg/kg IV) were also administered. The dog was discharged 48 h post-operatively with oral acetaminophen (11 mg/kg q 8 h), gabapentin (10 mg/kg q 8 h PO) and meloxicam (0.1 mg/kg q 24 h). Instructions to feed only canned or softened kibble and to avoid toys or mouth play were given to the owners.

The patient returned 2 days post-operatively and a small amount of mucoid discharge from the rostral skin incision was noted. The client was instructed to give oral amoxicillin/clavulanic acid (13.75 mg/kg q 12 h). At the time of oral surgery recheck and skin suture removal 14 days post-operatively the owners reported the dog was doing very well at home, eating and drinking well and was not experiencing difficulty breathing. However, occasional “hacking” had been observed. The extraoral closure site and the rostral extent of the oral incision was healed. However, an ~2 cm diameter area of dehiscence was noted at the caudal most extent of the intraoral closure site. The owners were advised that a definitive repair of the resultant oronasal fistula would be delayed until inflammation in all tissues had resolved. At 13 weeks post-operatively the client reported the development of a purulent draining tract on the face at the previous surgical site. Physical examination of the patient at 14 weeks post-operatively confirmed a purulent draining tract at the level of the medial canthus of the exenteration site. At 16 weeks post-operatively the patient was anesthetized for examination, which revealed communication of the facial draining tract with the previous surgery site and a concurrent oronasal fistula. Samples were taken from the draining tract for culture and sensitivity prior to thorough lavage and debridement. The patient was discharged with instructions to continue the previously prescribed amoxicillin/clavulanic acid and meloxicam pending culture and sensitivity results, which yielded moderate growth of methicillin resistant *Staphylococcus pseudointermedius* (MRSP). The patient was instructed to stop the prescribed amoxicillin/clavulanic acid and was provided a 2-week course of oral doxycycline (6 mg/kg q12 h) and metronidazole (10 mg/kg q12 h) as per the results of sensitivity testing. The patient presented at 23 weeks post-operatively for definitive surgery to close the oronasal fistula. An island axial pattern flap of the left hard palate was utilized to close the previously described defect. The patient was discharged with oral meloxicam (0.1 mg/kg q24 h) and gabapentin (13 mg/kg q8 h) for 14 days. The patient presented 4 weeks after revision surgery reportedly doing well at home. Oral examination confirmed healing of the previously repaired oronasal fistula site. Exposed palatal bone that was left to heal by second intention showed minor signs of granulation. Concern for inhibition of healing from the previously diagnosed MRSP was communicated to the client and the patient was restarted on the previous antibiotic regimen. The patient was reassessed by his primary care practitioner 5 weeks after revision surgery and photographs confirmed a near complete granulation bed at the open site. Follow up email contact with the client 13 weeks after revision surgery confirmed the patient was doing well at home with complete healing of the surgery site.

### Case 5

An 8-year-old spayed female vizsla was referred to the Medical Oncology service for evaluation of an osteosarcoma located inferior to the right eye. The referring veterinarian had previously extracted the right maxillary fourth premolar tooth and the right maxillary first molar tooth at the time of biopsy. On presentation, the visible mass measured 2.7 cm in length and was firm and non-mobile. Fine needle aspirates of the mandibular lymph nodes revealed no evidence of local metastasis. Thoracic CT revealed a partially mineralized 0.4 cm nodule in the left cranial lung lobe of unknown significance. Head CT revealed a 3.2 cm × 3.2 cm × 3.5 cm osteolytic and osteoproliferative soft tissue and mineral attenuating, moderately contrast enhancing mass within the right caudal maxilla. The mass extended caudally into the right pterygopalatine recess and orbital space causing dorsolateral displacement of the right globe and caudoventral displacement of the right zygomatic salivary gland. The mass extended rostrally and ventrally to include the alveolar bone associated with the previously extracted right maxillary fourth premolar tooth.

The case was referred to the Dentistry and Oromaxillofacial Surgery service for surgical treatment. The tumor was resected as an en bloc excision of the OZMC and associated musculature, oral mucosa, gingiva and dentition utilizing a combined intra- and extraoral approach with a transpalpebral exenteration. The osseous excision extended from the furcation of the right maxillary second premolar tooth to the mid-zygoma. The medial extent of the excision was the orbital part of the frontal bone and 2 mm to the contralateral side of the median palatine raphe. The excision including portions of the caudal maxilla, lacrimal, frontal, palatine, zygomatic, temporal, and pterygoid bones. Following excision, the wound was closed as described above.

The dog recovered uneventfully from general anesthesia and was managed overnight on CRI of fentanyl (2–5 μg/kg/hr) and ketamine (2–4 μg/kg/min) and an injection of carprofen (4.4 mg/kg SQ). A transdermal fentanyl patch (75 μg/hr) and piperacillin/tazobactam (45 mg/kg IV q 8 h) was also administered. The dog was discharged the following day with oral gabapentin (13 mg/kg q 8 h) and carprofen (4.4 mg/kg q 24 h). A nylon stockinette, and a loosely fitted tape muzzle was also applied to limit oral range of motion and tension on the intraoral surgery site. Instructions to feed only canned food and to avoid mouth play were given to the owners.

Histological examination confirmed the diagnosis of osteosarcoma with a narrow margin (500 μm) at the caudal aspect of the excision. Four days post-operatively, the patient presented for evaluation of malodorous smell from the oral cavity and hyporexia. Examination revealed a 6 cm dehiscence at the most caudal aspect of the intraoral closure. There was also purulent discharge present at the extraoral closure. The patient was discharged with instructions to continue medications as previously prescribed. The client reported unwillingness to administer gabapentin due to the level of sedation of the patient. Amoxicillin/clavulanic acid (17 mg/kg PO q 12 h) and metronidazole (10 mg/kg PO q 12 h) was prescribed, empirically. Mirtazapine (0.7 mg/kg PO q 24 h) was also prescribed as an appetite stimulant. Oronasal fistula repair was planned to occur when local inflammation and infection had resolved.

The patient was assessed by a veterinary dental specialist at a separate institution 7 days post operatively where physical examination revealed dehiscence of the extraoral closure and complete dehiscence of the intraoral closure resulting in a maxillo-oronasal fistula. Given the nature of the dehiscence and the likelihood of pursuing radiation therapy, surgical repair was recommended ahead of the previously planned schedule.

Fourteen days post-operatively the patient presented for repair of the maxillo-oronasal fistula with the Dentistry and Oromaxillofacial Surgery service. Under general anesthesia, a right inferior alveolar nerve block was administered with 1.5 mg bupivacaine and 15 μg buprenorphine. The right mandibular molar teeth were routinely extracted to prevent occlusal trauma to the maxillary soft tissue. Samples from the fistula were collected and submitted for culture and sensitivity. The maxillo-oronasal fistula was thoroughly lavaged. A myofascial axial pattern flap based on the temporalis muscle was utilized for repair of the oronasal fistula as described by Cavanaugh et al. (11) The patient recovered from anesthesia uneventfully and was monitored in the critical care unit with a CRI of fentanyl (2–5 μg/kg/hr) and ketamine (2–4 μg/kg/min). A subcutaneous injection of carprofen (4.4 mg/kg q24 h) was also administered. Intravenous piperacillin/tazobactam (45 mg/kg q 6 h) and metronidazole (10 mg/kg q 12 h) were also administered and the surgical site was iced every 2–4 h while hospitalized. Culture and sensitivity reported a multi-drug resistant Enterococcus infection and antibiotics were adjusted accordingly (discontinued metronidazole administration, added 5 mg/kg marbofloxacin PO q 24 h).

Significant facial edema developed post-operatively, which resolved by the fourth post-operative day. However, the patient was intermittently anorexic and the owners were unable to provide medications orally. The patient was re-admitted to the hospital 8 days following the revision surgery. An area of eschar overlying the rostral aspect of the extra-oral incision was debrided under sedation revealing a 3 cm diameter cutaneous dehiscence with exposure of the underlying temporalis muscle. The wound was thoroughly lavaged and cleansed with dilute chlorhexidine and sterile saline. Nylon (Dermilon, Covidien) sutures were placed around the periphery of the wound to allow for a tie-over bandage to be placed. An absorbable, antimicrobial polymeric wound matrix was used for direct wound dressing followed by sterile gauze and a moisture-protective barrier superficially. The bandage was held in place with umbilical tape. An esophagostomy tube was routinely placed during a short general anesthetic event the day prior to discharge to allow for administration of all oral medications. The patient was discharged with a new transdermal fentanyl patch (75 μg /hr) and oral carprofen (2.2 mg/kg q 12 h), acetaminophen (10 mg/kg q 12 h), amoxicillin/clavulanic acid (17 mg/kg q 12 h), marbofloxacin (5 mg/kg q 24 h) and ondansetron (2 mg/kg q 12 h). All medications were instructed to be administered via the esophagostomy tube, but the patient was to be allowed and encouraged to eat canned food. The owner was instructed to change the tie-over bandaging materials every 3 days.

The patient re-presented to the Dentistry and Oromaxillofacial Surgery service 20 days after the revision surgery. Orofacial examination revealed a layer of healthy granulation tissue over the dermal dehiscence site. Additionally, there was approximately 80% epithelialization of the intra-oral myofascial flap and no evidence of a persistent oral nasal fistula on conscious oral exam. The esophagostomy tube was removed and the sutures previously placed for the tie-over bandage were also removed. Surgical closure of the extra-oral dehiscence was discussed with the owner, but healing by second intention was elected. A soft food diet was recommended for an additional 3 weeks.

Six weeks following revision surgery, the patient presented to the medical oncology service to discuss adjunctive therapy options. Moderate restricted range of motion was identified when opening the patient's mouth. The patient was placed under general anesthesia for thorough oral examination and head CT which revealed a communication between the oropharynx and nasopharynx measuring ~1 cm in diameter, which was located at the junction of the myofascial flap and the soft palate. The client reported no clinical signs associated with the fistulation. Given the non-clinical nature of the fistula, surgical repair was not pursued. With narrow histologic tumor margins and the pulmonary nodule identified on thoracic CT, radiation therapy and chemotherapy were recommended, but not pursued by the owner.

## Discussion

In the report conveyed here, we have described a surgical technique to resect extensive OZMC tumors in which an intraoral approach is combined with a horizontal extraoral incision along the dorsolateral muzzle and zygoma that also incorporates a transpalpebral approach to orbital exenteration. This approach is a modification of a previously described combined intra- and extra-oral approach in order to access tumors of the caudal maxilla, in which the extraoral incision is made ventral to the globe (6).

**Figure 11:**
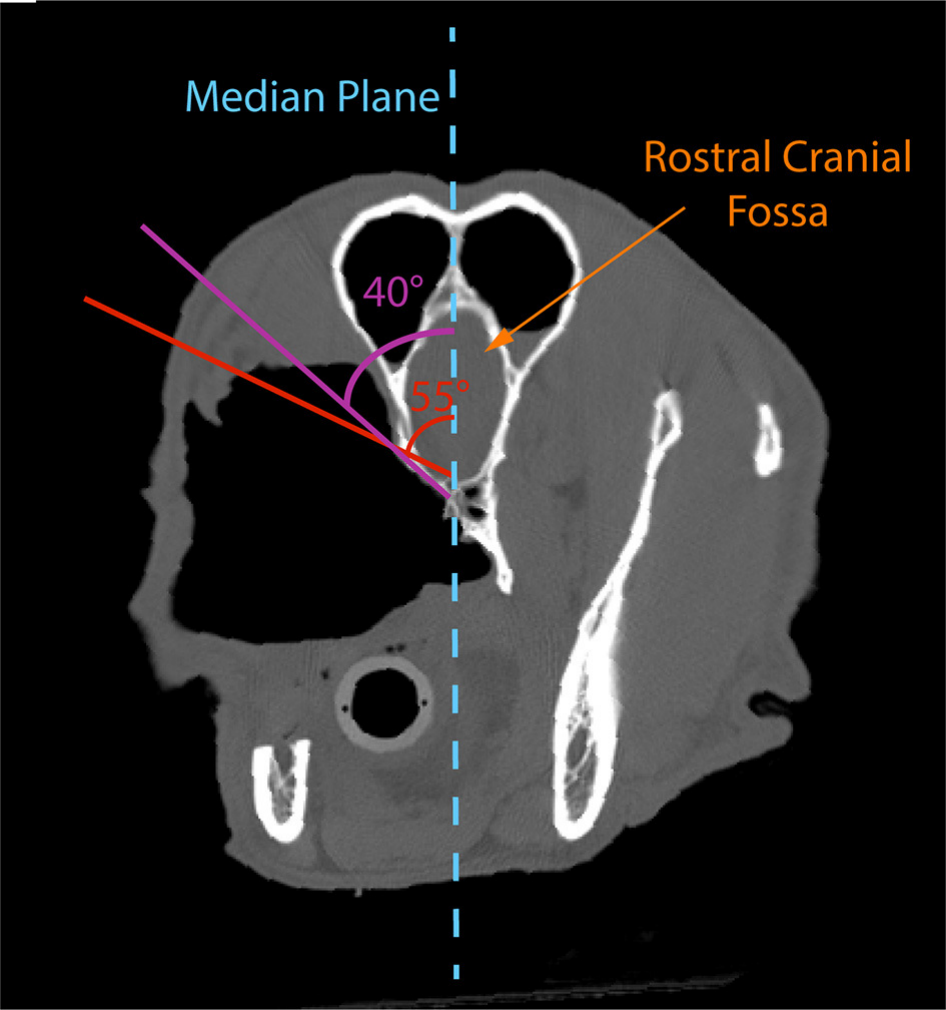
Postoperative axial CT image of case #5 depicting the need to make the medial orbitotomy at an angle of ~40° to the median plane. An alteration of as little as 15° (55° to the median plane) would direct the saw blade into the rostral olfactory bulb, which could lead to significant neurological complications.

Excision of the extensive OZMC tumors in this series included the entire inferior orbit and significant portions of the medial orbit. The medial orbit osteotomy was generally along a line beginning rostrally in the orbital crest of the lacrimal bone, just superior to the fossa of the lacrimal sac; coursing caudally along a line just inferior to the inferior orbital crest and optic canal; terminating just caudal to the hamulus of the pterygoid bone. The angle of approach (~40° from the median plane) for the medial orbitectomy must also be exact to ensure communication with the intraoral palatal approach and the caudal nasopharynx rather than penetrating into the rostral cranial fossa. An alteration of as little as 15° would direct the saw blade into the cerebrum, which could lead to significant neurological complications (12–14) (Figure 11).

In addition, maintaining the globe without orbital reconstruction may result in enophthalmos and resultant significant ocular complications (1). Diplopia (i.e. double vision) can occur secondary to malposition of a globe that leads to failure to fuse visual images (1). In humans, globe malposition has been reported to stimulate vagal activity from activation of the oculocardiac reflex resulting in significant headache, vertigo and severe nausea (2, 3). Entropion occurs secondary to an increase in orbital volume, as may be expected in facial fractures and orbitectomy and can lead to blepharospasm, conjunctival hyperemia, epiphora, mucoid ocular discharge, corneal ulceration, corneal scarring and vision loss (4, 5).

Visualization is vital to successful complete excision of OZMC tumors. Previous literature indicates that invasive orbital and periorbital tumors are often inadequately excised with local recurrence or persistence occurring in 36.7% of cases (15). In order to avoid the aforementioned ocular complications and achieve excellent visualization, we elected to perform orbital exenteration. By incorporating the extraoral skin incision with the transpalpebral approach to exenteration, we were able to accomplish the exenteration and OZMC excision with a singular extraoral incision, which may improve cosmetic outcomes and operative time.

Exenteration as part of the surgical plan for these patients was further supported by the possible need for post-operative radiation therapy. Commonly reported ocular-related radiation side effects are conjunctivitis, alopecia, keratoconjunctivitis sicca, erythema, uveitis and ulceration (16–18). Keratoconjunctivitis has been reported in more than a quarter of dogs undergoing radiation therapy of this site (18). Acute ocular side effects have a higher incidence with the use of curative radiation protocols and are less prevalent with the use of palliative radiation protocols (19). However, over 80% of patients in one study had ocular anomalies following radiation therapy and half of the canine patients had three or more ocular anomalies (19). Additionally, even with lower doses, histological evidence of irreversible long-term retinal changes has been reported (18). Given the risk of both acute and chronic ocular disease in these patients should post-surgical radiation be required, exenteration as part of a definitive surgery was elected.

The decision to exenterate the globe may be debatable. Highly conformal intensity modulated and image guided forms of radiotherapy have been successfully utilized to spare the globe from severe radiation toxicity in the setting of full-course definitive-intent radiotherapy in dogs with maxillofacial tumors. On the other hand, at our institution, stereotactic radiation therapy is selected on a case by case basis and utilized in the gross disease setting *only*, not to treat microscopic disease after surgery. Stereotactic radiation therapy is not recommended following surgery due to the concern of high doses of radiation to normal tissues especially to nerves, bone, and ocular structures. In the population of patients discussed here, definitive radiation therapy would be recommended following surgery.

In the future, we may consider sparing the globe in the few cases in which orbital reconstruction is pursued and exenteration is not necessary to achieve a medial orbitectomy. Orbital reconstruction would be necessary to avoid complications related to diplopia. Orbital reconstruction showed promising results in one case series (20). Short-term side effects consisted primarily of periocular swelling. Short and long-term follow-up revealed all eyes remained visual, with normal position, alignment and movement. However, detecting the effects of diplopia in dogs is challenging. Additionally, in our cases, surgical oncology tenets and need for precision mostly dictated orbital exenteration.

The major complication seen in these patients was significant intraoral incision line tension, which led to dehiscence and persistent oronasal fistula in three out of the five cases. The tension seen in these patients was not unexpected given that a significant amount of alveolar and palatal mucosa was excised as part of the excision. Tension was also related to the medial extent of removed tissue, often to or across the median palatine raphe. Taken with limited buccal mucosa, the lack of palatal mucoperiosteum made the reconstruction of an oral vestibule quite challenging. The dehiscence rate in this small case series (60%) is higher than previous reports in maxillectomies of 5–33% (7), likely due to the reasons described above. It is also reasonable to assume the location of these tumors played a role in dehiscence. Previous reports state that the rate of dehiscence is directly proportional to the caudal extent of the tumors, with up to 80% of reported post-operative dehiscence located caudal to the canine tooth (7). Additionally, the excision volumes of the cases presented here were large and likely represent the upper end of a normal distribution. Thus, the inherent risk of tension in this case series was considered pre-operatively and, in most patients, the occluding mandibular molar teeth were prophylactically extracted to reduce tension on the intraoral incisions. Surgical sites were closed with subdermal plexus advancement flaps. Other axial pattern flaps such as the angularis oris (21) or the superficial cervical axial pattern (22) flaps may have been considered. However, these flaps inherently possess concurrent complications that should not be dismissed. Ultimately, we elected to use the simplest flap design to achieve the objective. This principal is based on the concept that more advanced flaps should be reserved for the uncertain event of a dehiscence. In cases 2 and 4, an island axial pattern flap based on the contralateral major palatine artery was used to close the defect (23). In case 5, a myofascial axial pattern flap based on the ipsilateral temporalis artery was used (11). In all cases, the revision surgeries were successful at closing the dehiscence and resultant oronasal and maxillo-oronasal fistulas.

In order to improve visualization and access to the caudal oral cavity, a commissurotomy was also performed. It is conceivable that the proximity of the commissurotomy to the intra-oral mucosal incisions may have compromised the mucosal vascular supply and contributed to the risk of dehiscence. It was determined that the ability to achieve clean margins through optimal visualization was of paramount importance. As no patient experienced visible evidence of post-operative flap necrosis, the author's do not believe vascular supply was compromised. However, should one wish to use an angularis oris mucosal axial pattern flap to aid in intraoral closure, a commissurotomy would not be possible as it would likely damage the angularis oris artery. A plausible alternative would be to make a full-thickness, vertically oriented releasing incision through the superior labia to connect with the most rostral extent of the extra-oral incision; in effect, creating a full thickness labial flap that could be reflected caudally. This approach has been used for visualization of the OZMC in human oromaxillofacial surgery (24) and has been used to close wounds of the lip with a labial advancement flap in dogs (25). Such an approach should provide excellent visualization with the added benefit of preservation of the angularis oris artery should a mucosal axial pattern flap based on that artery become necessary for palatal reconstruction. To the author's knowledge this type of full-thickness labial flap used solely for improved access to the caudal oral cavity has not been described or evaluated in dogs.

While all our patients generally had minimal intra-operative hemorrhage, two patients did require a transfusion. Previous reports describe significant hemorrhage as the most common intra-operative complication seen in maxillofacial surgery (7). Hemorrhage was reported to be as high as 53% in other case series, with the majority of cases described as excessive to the point of needing transfusions (7). Hemorrhage has also been found to be significantly associated with larger and more caudally located tumors (7). Excessive hemorrhage could be expected given the location and size of the OZMC tumors of this case series, in which several large arteries are encountered during excision. It is the authors' opinion that the visualization achieved with the combined intra- and extra-oral approach coupled with exenteration and commissurotomy, as well as the use of piezosurgery for ostectomy, limited the amount of hemorrhage. Piezosurgery units have a higher frequency of vibration, 25–35 kHz, which allow cutting of mineralized tissue while sparing soft tissue; thereby, decreasing the likelihood of an inadvertent transection of a major vessel (26). Additional benefits of piezosurgery include decreased neurotrauma, increased visualization and decreased thermal injury to bone (26).

The surgical approach described here in which an orbital exenteration is used in conjunction with the dermal incision of a combined intra-and extraoral approach provides significant benefits for the excision of extensive OZMC tumors. Excellent visualization is obtained, common intra-operative complications are minimized and post-operative ocular complications are eliminated. The major complication, primarily caused by the size of the excision rather than the approach, is intra-oral wound dehiscence. This complication may be mitigated with the addition of axial pattern flaps (e.g., angularis oris mucosal or temporal myofascial axial pattern flaps) to avoid intra-oral wound tension.

## Data Availability Statement

The original contributions generated for the study are included in the article/supplementary material, further inquiries can be directed to the corresponding author/s.

## Ethics Statement

Ethical review and approval was not required for the animal study because the surgical procedures reported within this manuscript were performed in the course of providing a medically necessary procedure. The results are retrospective in nature and included clinical cases. Therefore, ethical review and approval was not required and is exempt from IACUC requirements. Written informed consent was obtained from the owners for all procedures performed.

## Author Contributions

AT, BR, and AG: drafting of case descriptions and discussion and final approval of version to be published. AT and JS: overall drafting of entire manuscript. JS: surgical concept and design, graphic development, interpretation, manuscript drafting, and final approval of version to be published. All authors contributed to the article and approved the submitted version.

## Conflict of Interest

The authors declare that the research was conducted in the absence of any commercial or financial relationships that could be construed as a potential conflict of interest.
